# The Effect of a Four-Month Low-Carbohydrate Diet on Visceral Adipose Tissue in Obese Subjects with Metabolic Dysfunction-Associated Steatotic Liver Disease (MASLD)

**DOI:** 10.3390/nu17172905

**Published:** 2025-09-08

**Authors:** Ornella Rotolo, Caterina Bonfiglio, Rosa Reddavide, Anna Maria Cisternino, Rosa Inguaggiato, Gianluigi Giannelli

**Affiliations:** 1 Clinical Trial Unit, National Institute of Gastroenterology IRCCS “Saverio de Bellis”, Castellana Grotte, 70013 Bari, Italy; 2 Unit of Data Science, National Institute of Gastroenterology IRCCS “Saverio de Bellis”, Castellana Grotte, 70013 Bari, Italy; catia.bonfiglio@irccsdebellis.it; 3 Center of Nutrition for the Research and the Care of Obesity and Metabolic Diseases, National Institute of Gastroenterology IRCCS “Saverio de Bellis”, Castellana Grotte, 70013 Bari, Italy; rosa.reddavide@irccsdebellis.it (R.R.); annamaria.cisternino@irccsdebellis.it (A.M.C.); rosa.inguaggiato@irccsdebellis.it (R.I.); 4 Scientific Direction, National Institute of Gastroenterology IRCCS “Saverio de Bellis”, Castellana Grotte, 70013 Bari, Italy; gianluigi.giannelli@irccsdebellis.it

**Keywords:** VAT, MASLD, obesity, diet

## Abstract

Background: Previous studies have shown a relationship between Visceral Adipose Tissue (VAT) and Hepatic Fat Content (HFC), and increases in HFC are linked to metabolic abnormalities similar to those associated with elevated VAT. Several short-term and long-term studies have supported these findings. Lifestyle interventions remain the cornerstone of treatment for Metabolic Dysfunction-Associated Steatotic Liver Disease (MASLD), although the ideal dietary regimen is still under debate. Methods: Data on 2040 patients were extracted from the Clinical Nutrition Unit database between 2017 and 2019. Of these, 474 subjects with MASLD and Body Mass Index (BMI) ≥ 35 kg/m^2^ were treated with a four-month low-carbohydrate dietary intervention called the “Strong Diet” (StD). VAT and liver stiffness were measured at baseline and after four months of treatment using ultrasound. Results: Our study demonstrates the significant efficacy of StD in reducing VAT in MASLD patients with moderate hepatic steatosis. In subjects with severe steatosis, there is no statistically significant response to dietary intervention. This may be attributed to several irreversible molecular mechanisms that fundamentally alter the hepatic microenvironment and limit the liver’s capacity for regeneration and metabolic recovery. Conclusions: Improvements were largely confined to patients with moderate MASLD, with limited benefit in severe disease. Although dietary intervention remains the cornerstone of MASLD management, patients with severe steatosis should be informed about the potential limited resolution of steatosis, even with optimal metabolic control.

## 1. Introduction

Non-alcoholic fatty liver disease (NAFLD) is one of the most prevalent causes of chronic liver disease, featuring a spectrum of liver damage that can progress to severe conditions, such as cirrhosis and hepatocellular carcinoma [1].

Both adults and children with fatty liver show abnormalities in glucose and lipid metabolism, making NAFLD a recognized part of the metabolic syndrome (MetS). The liver damage in NAFLD is believed to occur through a “multiple-hit process”. The first “hit” involves increased fat accumulation in the liver, followed by various factors that trigger inflammation [2]. In the early stages of NAFLD, triglyceride buildup in the liver is prominent. This condition is closely associated with insulin resistance, which is significantly affected by hypercaloric diets, sedentary lifestyles, and genetics. Fat accumulation in the liver causes lipotoxic damage to hepatocytes, driven by elevated levels of free fatty acids, free cholesterol, and other lipid metabolites. This results in mitochondrial dysfunction, oxidative stress, and stress related to the endoplasmic reticulum [3].

In overweight or obese subjects, the distribution of fat plays a more significant role than total body weight in determining metabolic disturbances that contribute to comorbidities shown to determine the obesity-related health impact [4].

In recent years, the understanding of non-alcoholic fatty liver disease has undergone a major shift in both terminology and concept. The term NAFLD has increasingly been replaced by Metabolic Dysfunction-Associated Steatotic Liver Disease (MASLD), following an international consensus statement from 2023 [5]. This change signifies a deeper insight into the disease’s development and clinical relevance, moving the focus from merely excluding significant alcohol intake to highlighting metabolic dysfunction as the key factor [6].

MASLD is a complex liver condition marked by the abnormal buildup of triglycerides in liver cells without significant alcohol use [7]. The revised definition sets out clearer diagnostic criteria and underscores its link with metabolic risk factors, like obesity, insulin resistance, dyslipidemia, and hypertension [8], offering a more precise understanding of its underlying mechanisms.

If left untreated, MASLD can develop into more serious liver conditions, including fibrosis and cirrhosis. MASLD has become the most prevalent cause of chronic liver disease worldwide, now representing a significant public health challenge. Early detection and management of this condition are essential to prevent severe liver complications [9]. The accumulation of VAT is closely associated with increased peripheral insulin resistance, often accompanied by a systemic, low-grade chronic inflammatory state known as lipoinflammation [10].

VAT, owing to its important anatomical location, delivers free fatty acids (FFAs) and adipokines directly to the liver via the portal vein. Sogabe et al.’s study identified visceral fat as a significant indicator of NAFLD in women with metabolic syndrome [10].

Similarly, research by Bouchi et al. demonstrated that higher levels of visceral fat strongly predict NAFLD in individuals with diabetes, including those with a normal body weight [11].

The “Carbohydrate-Insulin Model” of obesity proposes that consuming large amounts of processed carbohydrates triggers hormonal changes, particularly increased insulin secretion, which promotes fat storage, increases hunger, and reduces energy expenditure [12].

By increasing glucose uptake, reducing the release of free fatty acids from adipose tissue, and promoting fat and glycogen synthesis, postprandial hyperinsulinemia further stimulates appetite and contributes to weight gain [13]. Studies in animal models have demonstrated several benefits of Low-Carbohydrate Diets (LCDs), particularly those with a low glycemic index, compared to High-Carbohydrate Diets (HCDs) [14,15]. Research indicates that simply restricting energy intake on a high glycemic index diet does not prevent weight gain or increases in blood lipids and glucose [14]. On the other hand, an LCD has been shown to boost energy expenditure and aid in weight loss [15]. An early comparison of hypocaloric diets with different carbohydrate levels (LCDs versus HCDs) revealed that Very Low-Calorie Diets (VLCDs) [14] caused a more significant short-term decrease in intrahepatic lipid content (IHLC). However, after about 7% weight loss, IHLC levels became similar in both diet groups, with insulin sensitivity improvements remaining long-lasting [15].

A notable study by Gepner et al. demonstrated that a hypocaloric LCD combined with a Mediterranean Diet (MED), with or without physical activity (PA), resulted in the most substantial reductions in Visceral Adipose Tissue and IHLC compared to a hypocaloric low-fat diet (LFD). Interestingly, this effect was observed despite only moderate weight loss, implying that weight reduction alone may not fully capture the benefits of an LCD. Additionally, the decrease in IHLC was similar among participants with different levels of PA, highlighting the significant role of diet in influencing this outcome [16]. In this study, we explore the effect of a Low-Carbohydrate Diet, called the “Strong Diet” (StD), on Visceral Adipose Tissue (VAT) in MASLD patients.

## 2. Materials and Methods

### 2.1. Participant Selection

This research was conducted by the Clinical Nutrition Research Center for Obesity and Metabolic Diseases at the National Institute of Gastroenterology “Saverio de Bellis” in Castellana Grotte, Bari, Italy.

**Figure 1:**
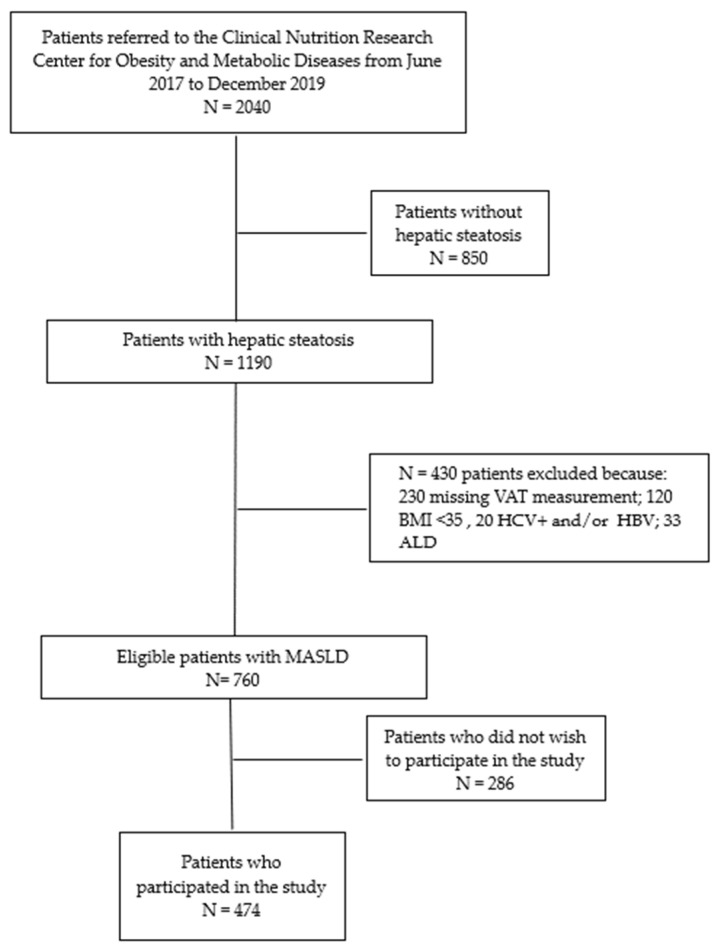
Flowchart of studies and participants. ALD: Alcoholic Liver Disease.

Among a total of 2040 patients referred from June 2017 to December 2019, we considered in this study 474 MASLD subjects who had hepatic steatosis, categorized as obese based on anthropometric and bioimpedance evaluations and clinical history assessments and who had completed the four-month dietetic protocol, called the “Strong Diet” (see Figure 1).

The study was approved by the local Medical Ethics Committee (Prot. Nr 92, dated 17 May 2017) and conducted in accordance with the ethical standards outlined in the 1964 Declaration of Helsinki. All participants provided written informed consent prior to enrolment.

### 2.2. Blood Test, Indirect Calorimetry, Anthropometric Measurements, and Bioelectrical Impedance Analysis

At the start of the study, all participants were examined between 8:00 and 11:00 a.m. after fasting overnight and underwent the following blood tests while still fasting: triglycerides (TGs), Total Cholesterol (TC), High-Density Lipoprotein Cholesterol (HDL), Gamma Glutamyl Transpeptidase (GGT), Alanine Aminotransferase (ALT), glycaemia, insulin, C-Reactive Protein (CRP), liver ultrasound, indirect calorimetry, bioelectrical impedance analysis, and anthropometric measurements (neck circumference, wrist circumference, waist circumference, hip circumference, WHR). Blood analyses were performed using the COBAS 8000 autoanalyser (ROCHE Diagnostic SPA, Monza, Italy).

Insulin resistance was estimated using the Homeostasis Model Assessment of Insulin Resistance (HOMA-IR), calculated by the following formula:

HOMA-IR = FSG (mg/dL) × fasting insulin (μIU/mL)/405

The baseline visit included a clinical and nutritional interview to record demographic information, risk factors, comorbidities, and dietary information. Body weight was measured to the nearest 0.1 kg using a digital scale (Seca 808, Hamburg, Germany; 0.1 kg accuracy), with participants wearing light clothes during assessment. Neck circumference, wrist circumference, waist circumference, and hip circumference were measured with a professional meter. Standing height of participants was measured to the nearest 0.5 cm, without shoes, using a wall stadiometer (Seca) and standard procedures. BMI was calculated as weight (kg)/height (m^2^).

Fat mass, lean mass, and muscle mass were estimated using bioelectrical impedance analysis (Akern, model Nutrilab, Florence, Italy). Patients were asked to avoid consuming alcoholic beverages 24 h before the test and to refrain from performing physical exercise 12 h beforehand. Participants were advised to empty their bladder 30 min before the test and to remove metal objects.

### 2.3. Ultrasound Examination of Fatty Liver

All subjects underwent a standardized ultrasound examination with a Hitachi H21 Vision (Hitachi Medical Corporation, Tokyo, Japan) at the start of the study and after four months. The visible liver parenchyma was examined using a 3.5 MHz transducer. A semi-quantitative scoring system was employed to assess liver fat content [17].

The degree of hepatic fat infiltration was evaluated based on liver echo texture, echo penetration, clarity of hepatic blood vessels, and differentiation of the liver diaphragm. Each criterion was scored to indicate fatty liver infiltration as follows:

- Score of 2 signified definite positive fatty liver;
- Score of 1 indicated probable positive fatty liver;
- Score of 0 denoted negative for fatty liver.

The total score, calculated by adding all three criteria, served as an indicator of fatty infiltration severity. The total score ranged from 0 to 6 and was classified as follows:

- 1–2: mild fat infiltration;
- 3–5: moderate fat infiltration;
- 6: severe fat infiltration;
- 0: no fatty liver present.

Additionally, in all participants, the thickness of Subcutaneous Abdominal Adipose Tissue (SAT) and VAT was measured with standardized ultrasound. SAT was measured in an epigastric transverse scan along the midline of the xypho-umbilical area. VAT was defined as the distance between the posterior abdominal wall surface and the anterior wall of the abdominal aorta. All measurements were recorded in millimeters.

### 2.4. Diet Intervention

Before the start of the diet intervention, each participant completed a 7-day dietary record, analyzed using a nutrition-specific database.

A Strong Diet consists of a combination of two Low-Carbohydrate Diets. The first diet is a regimen offering a very low-carbohydrate intake, 25% from carbohydrates, 25% from proteins, and 50% from lipids, and it is carried out for the first month of intervention. The second diet provides for the addition of a rate portion of whole bread or rolled oats at breakfast and a rate portion of whole pasta or grain once or twice a week at lunch, so that the percentages of macronutrients are 40% from carbohydrates, 25% from proteins, and 35% from lipids. More specifically, the diet carried out during the first month is made up as follows: breakfast with yogurt or eggs plus fresh and local fruit; snacks with nuts (walnuts and/or almonds, not grounded nor salted); and lunch and dinner with fresh and local vegetables—raw or cooked—seasoned with extra virgin olive oil plus a serving of a protein source (pulses, poultry, seafood/fish, eggs, dairy, red meat). Protein sources had to vary according to the frequency recommended in the Mediterranean Diet Pyramid. For the remaining three months of intervention, participants added a serving of whole bread or rolled oats to breakfast and ate a serving of whole pasta, rice, or other types of cereal (barley, spelt, oat) 1–2 times a week, as a main course, instead of a protein source. The caloric amount of the diet assigned to every patient (BMI ≥ 35) was assessed by performing indirect calorimetry (COSMED, Quark RMR, Albano Laziale—Rome, Italy) after overnight fasting and considering the value of the resulting REE (Resting Energy Expenditure, Kcal/day). The caloric intake ranged from 1200 to 2000 kcal/day. For the single subject, the calorie intake did not vary after the first month of intervention, with the introduction of the starchy meal. All participants had a food diary to record the food they consumed daily. Every two months from the baseline visit, one-on-one counseling with a dietitian or nutritionist was conducted to analyze food diary recording to assess compliance with the diet or any adverse event. Each patient met the same dietitian or nutritionist in order to avoid bias.

The food diary also included a section dedicated to physical activity. Each day, patients reported the amount of time spent sitting, expressed in hours. Additionally, in the section on physical activity, participants indicated the type of activity they had engaged in during the day, such as whether they had gone to the gym or taken a walk. After four months of dietary treatment, the project participants underwent repeat blood tests, bioelectrical impedance analysis, anthropometric measurements, and liver ultrasound scans.

### 2.5. Statistical Analysis

Follow-up time and biological variables are presented as mean (±SD) for continuous variables and as frequencies (%) for categorical variables. We used the Wilcoxon matched-pairs test for continuous variables to compare two groups, which is the non-parametric equivalent of the paired *t*-test, suitable when data are not normally distributed. For categorical variables, the χ2 test was employed to assess differences.

A Generalized Estimating Equation (GEE) [18] was utilized to estimate the longitudinal trajectories of VAT. Since the outcome variables were not normally distributed, a gamma distribution (using an identity link) was assumed, with an unstructured correlation matrix to handle the data.

GEE models are especially valuable in biomedical research for estimating mean changes in biomarker levels while controlling for covariates, accounting for correlations among repeated measurements within subjects.

In selecting covariates for the model, variables already linked to MASLD, such as BMI, waist circumference, HDL cholesterol, triglycerides, fasting glucose, and hypertension, were excluded.

Subsequently, predictor variables were chosen based on the existing literature, and the minimum absolute reduction and selection (LASSO) [19] procedure was applied to narrow down candidate predictors and pinpoint the most relevant for the model.

Additionally, the variance inflation factor (VIF) was evaluated to detect multicollinearity, leading to the exclusion of confounders with VIF > 5 [20]. The final model included gender, age, NAFLD severity, WHR, GGT, ALT, and HOMA as covariates to assess the effect of diet-induced VAT reduction and the influence of these factors.

Outcome predictions were obtained through post-estimation tools, such as contrasts of marginal linear predictions and predictive margins, with the results graphically displayed on the natural VAT measurement scale.

Statistical significance was determined by 95% confidence intervals (CIs) for *p*-values < 0.05. A retrospective power analysis confirmed that our sample size was sufficient, ensuring the reliability of the findings. All analyses were conducted using Stata software v. 19.0. (Stata Corp, 4905 Lakeway Drive, College Station, TX 77845, USA).

## 3. Results

Among a total of 2040 patients referred from June 2017 to December 2019, we considered 474 MASLD subjects who had hepatic steatosis and were categorized as obese based on anthropometric and bioimpedance evaluations, clinical history assessments, and laboratory analyses, and who had completed the four-month StD.

Of this population, 68.78% were female (N = 326) and 49.47% had a BMI greater than 40 (N = 234). The mean age of patients was 56.55 years (±12.63): 56.79 years (±12.61) for females and 56.03 years (±12.71) for males.

Of the 474 patients, 65 (13.7%) were diabetic, 242 (51.1%) suffered from hypertension, 76 (16.0%) from dyslipidemia, 43 (9.1%) from hypothyroidism, and 16 (3.4%) from depression. Additionally, 69 (14.6%) reported engaging in physical activity, 36 of them walking.

Table 1 shows the study sample’s anthropometric, metabolic, and body composition parameters before and after the StD. After four months on the StD, VAT decreased by 25%, from 73.98 (49.46) to 55.74 (53.86).

The data shown in Table 1 indicate statistically significant improvements for all blood and anthropometric values.

Regarding MASLD, a reduction in steatosis severity was observed: patients with severe steatosis decreased from 155 to 56, those with moderate from 146 to 155, and those with mild from 111 to 200, while 63 patients no longer had steatosis (see Table 1).

There was also a decrease in the number of patients in the last two BMI categories between pre- and post-diet and an increase in the first three (see Table 1).

Table 2 shows the results of the Generalized Estimating Equation (GEE). Among subjects given the StD, there was a statistically significant effect of time for VAT (β: −11.43, 95% CI: −17.44; −5.41). As to the severity of NAFLD, there was a statistically significant downward trend in VAT when comparing it with severe NAFLD: −15.62 (95% CI: −27.64; −3.67) for moderate, −21.75 (95% CI: −34.38; −9.12) for mild, and −34.58 (95% CI: −51.52; −17.65) in subjects no longer with steatosis.

In other words, over the four months of dietary treatment, the value of VAT was reduced by an average of 11.43 cm^3^. Analyzing the degree of NAFLD, using severe steatosis as the comparison category, a reduction in VAT of 15.62 cm^3^ was observed in the moderate group at the end of the four months, a decrease of 21.75 cm^3^ in the mild group, and among those who no longer had steatosis, a reduction in VAT of 34.58 cm^3^ was found (see Table 2).

Table 3 illustrates the contrasts between the expected mean values for the effects of each NAFLD grade over the four months of StD treatment.

**Figure 2:**
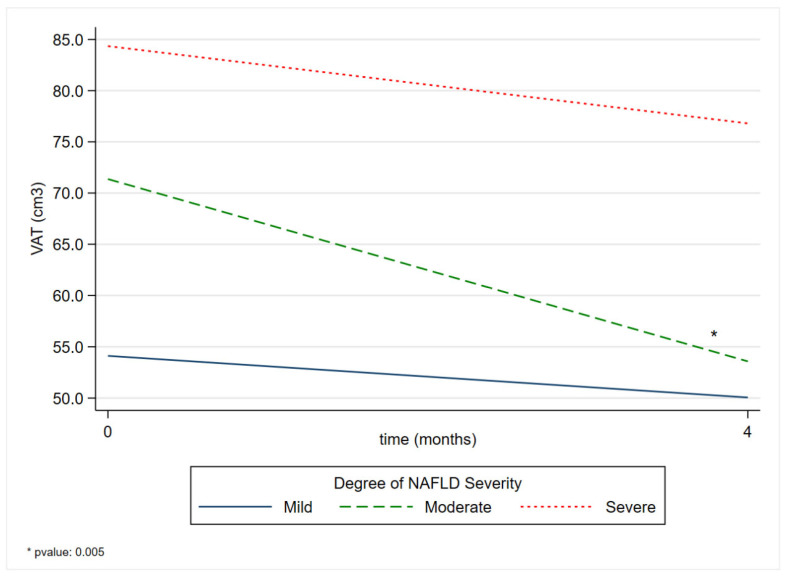
Comparison of VAT margins between the degree of NAFLD severity before and after a four-month Strong Diet. VAT: Visceral Adipose Tissue; NAFLD: non-alcoholic fatty liver disease.

The comparison between time and NAFLD severity grade showed a reduction in severity across all NAFLD grades. Only in patients with moderate steatosis did this comparison reveal a statistically significant reduction in VAT. From the start of dietary treatment to four months later, a reduction of 16.70 cm^3^ [*p*-value 0.005 (95% CI −28.44, −4.96)] was observed (Table 3), as shown in Figure 2.

The margins presented in Table S1 represent the averages derived from the predictions of the adjusted model. A decrease in the mean predicted VAT over time was observed in all grades of NAFLD severity.

## 4. Discussion

### 4.1. Effectiveness of StD on VAT Reduction

The dietetic protocol, StD, consists of a combination of two diets, which both offer a low-carbohydrate intake. The first diet is a regimen with a very low-carbohydrate content, to be observed for the first month. The second diet provides for the addition of a rate portion of whole bread or rolled oats at breakfast, and a rate portion of whole pasta or grain once or twice a week at lunch, to be observed for the remaining three months.

This diet features foods typical of the Mediterranean pattern. It restricts red and processed meats, added sugars like monosaccharides and disaccharides in foods and drinks, and natural sugars found in syrups, fruit juices, and concentrates. The diet includes fish, seafood, poultry, eggs, dairy, and walnuts or almonds, all characterized by a low glycemic index due to higher fiber intake from vegetables and whole grains.

Our study demonstrates the significant efficacy of a low-carbohydrate, four-month dietary intervention, StD, in reducing VAT in patients with MASLD. The mean VAT reduction of 25% (from 73.98 to 55.74 cm^3^) indicates a clinically relevant improvement that aligns with the international literature evidence identifying VAT as a key therapeutic target in managing non-alcoholic fatty liver disease [21].

Patients with moderate steatosis showed a strong and statistically significant VAT reduction.

Numerous studies support the benefits of a diet characterized by a low-carbohydrate intake [22] and by typical foods of the Mediterranean Diet [23]. As previously reported, a notable study by Gepner et al. [24] demonstrated that a hypocaloric LCD combined with an MED resulted in the most significant reductions in VAT and IHLC compared to a hypocaloric low-fat diet (LFD).

Baratta F. et al. demonstrated a notable inverse relationship between red and processed meat consumption and NAFLD [25,26].

Notably, this link appears to be primarily driven by animal protein, as no similar association has been observed with plant-based protein [27,28].

A compelling explanation for this phenomenon was proposed by Alferink et al., suggesting that the diet-induced acid load plays a key role [22].

In examining the link between the increasing intake of fructose and the rising rates of NAFLD and metabolic syndrome, fructose has been associated with insulin resistance (IR), hepatic lipid buildup, and hypertriglyceridemia, all of which contribute to the development of type 2 diabetes and cardiovascular diseases [23]. This connection occurs because ingested fructose undergoes first-pass metabolism in the liver. Fructose functions as a more direct substrate for de novo lipogenesis, resulting in greater intrahepatic lipid accumulation [29]. Additionally, unlike glucose metabolism, fructose-driven gluconeogenesis happens independently of insulin and cellular energy status [29], leading to ATP depletion and increased uric acid production, which further promotes oxidative stress and insulin resistance [30].

Therefore, beverages sweetened with fructose have been linked to increased de novo lipogenesis, dyslipidemia, and VAT accumulation and reduced insulin sensitivity [31]. A recent randomized controlled trial confirmed these findings, demonstrating that both fructose and sucrose led to an increased basal secretion rate of free fatty acids. This suggests a possible adaptive response to regular fructose intake from sugar-sweetened beverages [32]. Additionally, limiting fructose consumption was shown to reduce intrahepatic lipid content [33]. Similarly, sugar-sweetened beverages have been associated with a higher prevalence of NAFLD, the presence of non-alcoholic steatohepatitis (NASH), and even more advanced fibrosis [34].

The second diet, enriched with whole grains and characterized by a low glycemic index, was crucial in further reducing VAT related to the cardiometabolic risk profile. These grains contain phenolic compounds that boost antioxidant and anti-inflammatory effects [35]. Its benefits are mainly due to the high consumption of Monounsaturated Fatty Acids (MUFAs) from extra virgin olive oil, Polyunsaturated Fatty Acids (PUFAs) from fish and nuts, and fiber from fruits, legumes, and vegetables. Additionally, it involves consuming less Saturated Fatty Acids (SFAs) and hydrogenated fats. Increased intake of omega-3 PUFAs and MUFAs is beneficial because it reduces VAT-associated inflammation, improves endothelial dysfunction, and alleviates dyslipidemia [36].

Regular nut consumption may be associated with a lower risk of advanced fibrosis in NAFLD patients [37]. Despite their high caloric content, nuts are not linked to weight gain [38]. Conversely, their anti-inflammatory properties—such as the presence of omega-3 PUFAs—may help improve NAFLD [39]. Additionally, replacing packaged and processed foods with fresh foods reduces salt intake, which has been linked to a decreased risk of NAFLD [40]. In our study, patients with severe steatosis showed a reduction in VAT that was not statistically significant. This suggests that individuals with moderate metabolic impairment might benefit from a more noticeable effect of nutritional intervention, possibly due to a greater capacity for metabolic improvement in the absence of irreversible changes caused by fibrosis. Conversely, in individuals with severely impaired metabolic status, the lack of statistically significant response to dietary intervention can be attributed to several irreversible molecular mechanisms that fundamentally alter the hepatic microenvironment and restrict the liver’s ability to regenerate and recover metabolism [41].

The activation of Hepatic Stellate Cells (HSCs) is essential in transitioning from reversible steatosis to permanent fibrosis. Friedman et al. demonstrated that in advanced steatosis, the liver’s inflammatory environment becomes self-perpetuating, with immune cells, such as macrophages and T-lymphocytes, forming persistent inflammatory zones within fibrotic regions. These cells continuously release pro-fibrotic cytokines such as transforming growth factor-β (TGF-β), Platelet-Derived Growth Factor (PDGF), and various interleukins, which sustain HSC activation, even when the original metabolic triggers are absent [42].

Under normal physiological conditions, HSCs remain in a quiescent state, primarily serving as vitamin A storage cells. However, in response to chronic hepatic injury and inflammation associated with MASLD progression, these cells undergo a significant phenotypic transformation into an activated, myofibroblastic state [43]. Once activated, HSCs gain an increased proliferative capacity, heightened contractility, and most notably, the ability to produce large amounts of extracellular matrix proteins, especially collagen types I and III. This phenotypic switch becomes increasingly irreversible as fibrosis advances, with activated HSCs losing their capacity to revert to the quiescent state even when the initial inflammatory stimulus is removed or metabolic conditions improve through dietary intervention [43].

In conclusion, Mediterranean Diet foods appear to be responsible for improving VAT in patients with MASLD; in particular, these improvements were mainly limited to patients with moderate steatosis, with limited benefits in more severe cases.

### 4.2. Improvement in Hepatic Steatosis Grading

The improvement in hepatic steatosis observed in our study holds significant clinical relevance. The progression of 63 patients (13.3%) towards a complete absence of steatosis, along with a notable reduction in severe cases (from 155 to 56 patients), supports the effectiveness of the nutritional approach in MASLD regression. These findings align with studies by Vilar-Gomez et al. [3], which showed that a 7–10% weight loss can lead to meaningful histological improvements in NAFLD. GEE analysis indicated that patients with moderate steatosis experienced the most significant VAT reduction over time (−17.40 cm^3^, *p*-value = 0.003), suggesting that this subgroup may be especially responsive to nutritional intervention. This supports the idea that there is an optimal window for intervention during which the mechanisms for reversing steatosis are most actively engaged.

### 4.3. Impact on Anthropometric and Metabolic Parameters

The notable decrease in body weight (from 106.32 to 94.36 kg) and BMI (from 41.01 to 37.66 kg/m^2^) seen in our study demonstrates the effectiveness of the nutritional approach implemented. The average loss of 11.96 kg over 4 months amounts to a weight loss rate of roughly 3 kg/month, which aligns with the safe and effective range outlined by international guidelines [43,44].

### 4.4. Improvements in Hepatic and Metabolic Biomarkers

The significant improvements in ALT levels (from 34.18 to 23.50 U/L) and GGT (from 36.44 to 25.46 U/L) confirm the effectiveness of nutritional intervention in reducing hepatic inflammation. These findings align with studies by Yun-Huei Ko et al. [21] and Properzi et al. [45], which showed that reducing body weight and VAT is linked to parallel improvements in hepatic function. The enhancement in metabolic profile, demonstrated by significant reductions in triglycerides (from 146.08 to 106.06 mg/dL), glycaemia (from 109.39 to 98.58 mg/dL), and HOMA (from 5.14 to 3.05), indicates a systemic effect of the intervention that goes beyond the improvement of hepatic steatosis. These changes imply improved insulin sensitivity, a key factor in MASLD pathogenesis, as described by Younossi et al. [46].

## 5. Study Limitations

Our study presents some limitations that must be considered in results interpretation. The study design does not include a control group, which limits the possibility of attributing the observed improvements exclusively to the nutritional intervention.

However, this choice was approved by the Ethics Committee, considering the nature of the treatment and its feasibility in the clinical setting.

Additionally, hepatic steatosis assessment by ultrasound, while it is a widely used method in clinical practice that uses a standardized ultrasound report validated in several studies, presents precision limitations compared to more advanced techniques, such as magnetic resonance spectroscopy [47] or ultrasound-based elastography with FibroScan [48]. The absence of long-term follow-up data is a limitation, as the sustainability of the observed improvements remains uncertain and, although the reduction in VAT is promising, the connection with long-term outcomes (progression to cirrhosis, cardiovascular risk) cannot be confirmed.

## 6. Conclusions

In conclusion, our study demonstrates that four months of structured dietary intervention (StD) can significantly reduce VAT, especially in patients with MASLD and moderate hepatic steatosis. The improvements observed, particularly in patients with moderate steatosis (a stage in which the molecular mechanisms are not yet fully defined), support the use of targeted nutritional strategies as an effective therapeutic approach.

These molecular insights have significant implications for clinical practice and patient counseling. This precisely leads us to believe that it is important and necessary to intervene with lifestyle modifications as early as possible, certainly before steatosis undergoes its natural progression and cellular changes occur that lead to severe, untreatable steatosis.

Although dietary intervention remains the cornerstone of MASLD management, patients with severe steatosis should be informed about the potential limited resolution of steatosis, even with optimal metabolic control. Additionally, some genetic polymorphisms, not explored in this study, may contribute to the reduced improvement in patients with severe steatosis, but these speculations go beyond the scope of our work and certainly require further in-depth research and clarification.

## Figures and Tables

**Table 1:** Description of the whole sample at the assessment time (pre- and post-Strong Diet). All data are shown as mean (±SD) or percentage (%).

Variable ^a^	Baseline	4 Months	*p*-Value ^ε^
N	474	474	
VAT (cm^3^)	73.98 (49.46)	55.74 (53.86)	<0.001
MASLD (%)			
No	0 (0.00)	63 (13.3)	0.09
Yes	474 (100)	411 (86.7)	
Degree of NAFLD (%)			
Absent	0 (0.0)	63 (13.3)	<0.001
Mild	111 (26.9)	200 (42.2)	
Moderate	146 (35.4)	155 (32.7)	
Severe	155 (37.6)	56 (11.8)	
SAT (cm^3^)	27.73 (17.68)	20.99 (23.92)	<0.001
Age (years)	56.55 (12.63)		
Gender (%)			
Female	326 (68.8)		
Male	148 (31.2)		
Anthropometric Measurements			
Weight (kg)	106.32 (16.51)	94.36 (15.26)	<0.001
BMI (kg/m^2^)	41.01 (5.08)	37.66 (27.11)	0.008
Categories of BMI (%)			
<25	0 (0.0)	1 (0.2)	<0.001
25–29.9	0 (0.0)	24 (5.1)	
30–34.9	19 (4.0)	178 (37.6)	
35–40	220 (46.5)	168 (35.4)	
>40	234 (49.5)	103 (21.7)	
Waist circumference (cm)	122.31 (11.25)	111.75 (11.02)	<0.001
Hip circumference (cm)	129.66 (11.83)	121.17 (10.86)	<0.001
WHR	0.95 (0.08)	0.92 (0.07)	<0.001
Lean mass (Kg)	61.71 (12.10)	57.87 (14.22)	<0.001
Muscle mass (Kg)	35.18 (10.99)	20.87 (17.52)	<0.001
Fat mass (Kg)	44.20 (10.92)	34.03 (11.27)	<0.001
Blood test			
ALT (U/L)	34.18 (23.25)	23.50 (9.97)	<0.001
GGT (U/L)	36.44 (36.33)	25.46 (25.15)	<0.001
TC (mg/dL)	195.94 (37.17)	191.50 (96.70)	0.37
HDL (mg/dL)	50.47 (12.11)	52.73 (12.50)	0.012
TG (mg/dL)	146.08 (76.10)	106.06 (75.25)	<0.001
Glucose (mg/dL)	109.39 (31.81)	98.58 (19.80)	<0.001
Insulin (UI/mL)	18.85 (13.46)	12.17 (11.11)	<0.001
HOMA IR	5.14 (4.20)	3.05 (3.21)	<0.001
CRP (mg/L)	3.55 (4.94)	2.98 (4.20)	0.100

^a^ Mean ± (SD). Percentages calculated per column. TC: Total Cholesterol; VAT: Visceral Adipose Tissue; SAT: Subcutaneous Abdominal Adipose Tissue; NAFLD: non-alcoholic fatty liver disease; BMI: Body Mass Index; WHR: Waist-to-Hip ratio; TGs: triglycerides; TC: Total Cholesterol; HDL: High-Density Lipoprotein Cholesterol; GGT: Gamma Glutamyl Transpeptidase; ALT: Alanine Amino Transferase; Homa: Homeostatic Model Assessment; CRP: C-Reactive Protein; ^ε^ Wilcoxon matched-pairs test and Pearson’s chi-square tests were used to test differences between means and proportions, respectively.

**Table 2:** Generalized Estimating Equation (GEE): expected values for Visceral Adipose Thickness (VAT) according to the NAFLD degree of severity and follow-up time for StD.

VAT	β	95% CI	*p*-Value
**Time**			
Baseline	0		
4 months	−11.43	−17.44; −5.41	<0.001
**NAFLD degree of severity**			
Severe	0		
Moderate	−15.62	−27.64; −3.67	0.011
Mild	−21.75	−34.38; −9.12	0.001
Absent	−34.58	−51.52; −17.65	<0.001

Model adjusted for age, gender, WHR, GGT, ALT, and HOMA IR. WHR: Waist-to-Hip ratio; GGT: Gamma Glutamyl Transpeptidase; ALT: Alanine Amino Transferase; HOMA IR: Homeostatic Model Assessment; β: Regression Coefficient.

**Table 3:** Generalized Estimating Equation (GEE): contrasts the degree of NAFLD severity and time from baseline by StD.

Time # Degree of NAFLD Severity	Contrast	*p*-Value	95% CI
(4 vs. 0) # Severe	−7.25	0.443	−25.80, 11.29
(4 vs. 0) # Moderate	−16.70	0.005	−28.44, −4.96
(4 vs. 0) # Mild	−5.67	0.431	−19.78, 8.45
(4 vs. 0) # Absent	Not estimatable		

Model adjusted for age, gender, WHR, GGT, ALT, and HOMA IR. WHR: Waist-to-Hip ratio; GGT: Gamma Glutamyl Transpeptidase; ALT: Alanine Amino Transferase; HOMA IR: Homeostatic Model Assessment.

## Data Availability

The data presented in this study are openly available in http://doi.org/10.6084/m9.figshare.29891171, accessed on 5 September 2025.

## Supplementary Materials

The following supporting information can be downloaded at: https://www.mdpi.com/article/10.3390/nu17172905/s1, Table S1: Predicted means, with 95% confidence intervals, of VAT by degree of NAFLD severity and pre- and post-StD.

## Abbreviations

ALD	Alcoholic Liver Disease
ALT	Alanine Amino Transferase
ASA	Abdominal Subcutaneous Adipose
BMI	Body Mass Index
CRP	C-Reactive Protein
FFAs	Free Fatty Acids
GEE	Generalized Estimating Equation
GGT	Gamma Glutamyl Transpeptidase
HCD	High-Carbohydrate Diet
HDL	High-Density Lipoprotein Cholesterol
HFC	Hepatic Fat Content
HOMA IR	Homeostatic Model Assessment Insulin Resistance
HSCs	Hepatic Stellate Cells
IHLC	Intrahepatic Lipid Content
IR	Insulin Resistance
LCD	Low-Carbohydrate Diet
LFD	Hypocaloric Low-Fat Diet
MASLD	Metabolic Dysfunction-Associated Steatotic Liver Disease
MED	Mediterranean Diet
MUFAs	Monounsaturated Fatty Acids
NAFLD	Non-Alcoholic Fatty Liver Disease
NASH	Non-Alcoholic Steatohepatitis
PA	Physical Activity
PDGF	Platelet-Derived Growth Factor
PUFAs	Polyunsaturated Fatty Acids
SFAs	Saturated Fatty Acids
Std	Strong Diet
TC	Total Cholesterol
TGs	Triglycerides
TGF-Β	Growth Factor-Β
VAT	Visceral Adipose Tissue
VLCD	Very Low-Calorie Diet
WHR	Waist-to-Hip Ratio
