# Supplementary material for: The Effect of a Four-Month Low-Carbohydrate Diet on Visceral Adipose Tissue in Obese Subjects with Metabolic Dysfunction-Associated Steatotic Liver Disease (MASLD)
Source: Nutrients. 2025 Sep 8;17(17):2905. doi: 10.3390/nu17172905 (PMC12429932; doi:10.3390/nu17172905)
Supplement: Supplementary file 1 [file nutrients-17-02905-s001.zip › nutrients-3844869-supplementary.pdf]

Supplementary Materials

Table S1. Predicted means, with 95% Confidence Intervals, of VAT by Degree of NAFLD Severity and pre- and post StD.

VAT (cm^3)	Margins	95% CI
Degree of NAFLD#time:		
Severe#Baseline	83.96	75.82; 92.11
Severe#4 months	77.13	61.41; 92.86
Moderate#Baseline	71.30	62.31; 80.29
Moderate#4 months	53.90	47.31; 60.50
Mild#Baseline	53.80	41.71; 65.90
Mild#4 months	49.88	45.45; 54.31

Model adjusted for Age, Gender, WHR, GGT, ALT, and HOMA. WHR: Waist-to-Hip ratio; GGT: Gamma Glutamyl Transpeptidase, ALT: Alanine Amino Transferase, HOMA IR: Homeostatic Model Assessment; VAT: Visceral Adipose Tissue ; NAFLD: Non-Alcoholic Fatty Liver Disease.
